# The Structure of Gd^3+^-Doped Li_2_O and K_2_O Containing Aluminosilicate Glasses from Molecular Dynamics Simulations

**DOI:** 10.3390/ma14123265

**Published:** 2021-06-12

**Authors:** Mohamed Zekri, Andreas Herrmann, Andreas Erlebach, Kamel Damak, Christian Rüssel, Marek Sierka, Ramzi Maâlej

**Affiliations:** 1 Georesources Materials Environment and Global Changes Laboratory (GEOGLOB), Faculty of Sciences of Sfax, Sfax University, Sfax 3018, Tunisia; mohamed.zekri.etud@fss.usf.tn (M.Z.); kamel.damak@fss.rnu.tn (K.D.); ramzi.maalej@fss.usf.tn (R.M.); 2 Institute of Materials Science and Engineering, Ilmenau University of Technology, 98693 Ilmenau, Germany; andreas.herrmann@tu-ilmenau.de; 3 Otto Schott Institute of Materials Research, Friedrich Schiller University Jena, 07743 Jena, Germany; andreas.erlebach@natur.cuni.cz (A.E.); ccr@uni-jena.de (C.R.)

**Keywords:** glass, aluminosilicate, rare earth, gadolinium, atomistic simulations, glass structure

## Abstract

Understanding the atomic structure of glasses is critical for developing new generations of materials with important technical applications. In particular, the local environment of rare-earth ions and their distribution and clustering is of great relevance for applications of rare earth-containing glasses in photonic devices. In this work, the structure of Gd_2_O_3_ doped lithium and potassium aluminosilicate glasses is investigated as a function of their network modifier oxide (NMO–Li_2_O, K_2_O) to aluminum oxide ratio using molecular dynamics simulations. The applied simulation procedure yields a set of configurations, the so-called inherent structures, of the liquid state slightly above the glass transition temperature. The generation of a large set of inherent structures allows a statistical sampling of the medium-range order of the Gd^3+^ ions with less computational effort compared to other simulation methods. The resulting medium-range atomic structures of network former and modifier ions are in good agreement with experimental results and simulations of similar glasses. It was found that increasing NMO/Al ratio increases the network modifier coordination number with non-bridging oxygen sites and reduces the overall stability of the network structure. The fraction of non-bridging oxygen sites in the vicinity of Gd^3+^ ions increases considerably with decreasing field strength and increasing concentration of the network modifier ions. These correlations could be confirmed even if the simulation results of alkaline earth aluminosilicate glasses are added to the analysis. In addition, the structure predictions generally indicate a low driving force for the clustering of Gd^3+^. Here, network modifier ions of large ionic radii reduce the probability of Gd–O–Gd contacts.

## 1. Introduction

Aluminosilicate glasses based on alkali or alkaline earth aluminosilicates find a wide range of technical applications. They are used in the form of glass fibers for reinforced materials (mostly magnesium calcium aluminosilicate glasses) or as scratch-resistant display glasses based on the sodium aluminosilicate glass system [1]. The strength of sodium aluminosilicate glasses can be improved by applying an ion exchange process in molten KNO_3_, which results in a replacement of surface-near Na^+^ ions for K^+^ ions. The temperature supplied is much below the glass transition temperature and hence the replacement of Na^+^ by the much larger K^+^ induces compressive stresses close to the surface, which cannot relax. This results in a high tensile strength exceeding 800 MPa [2]. Alkali aluminosilicate glasses are also used for the preparation of glass-ceramics. Here, glass-ceramics with zero thermal expansion based on the Li_2_O/Al_2_O_3_/SiO_2_ system are particularly important. During thermal treatment, the crystallization of crystal phases such as β-quartz, spodumene, and eucryptite occurs which all exhibit a negative coefficient of thermal expansion [3]. Another important field of application of aluminosilicate glasses is high strength glass-ceramics in the system MgO/Al_2_O_3_/SiO_2_ [4,5]. According to recent reports, this glass system enables the preparation of glass ceramics with a tensile strength exceeding 1 GPa [6]. In addition, in the past few years, rare-earth-doped aluminosilicate glasses were investigated as laser materials. In comparison to other laser glasses, such as phosphate or fluoride phosphate glasses, they possess much more advantageous thermo-mechanical properties. Here, most important are the low coefficients of thermal expansion (CTE) but also their higher mechanical strength, their high hardness, and last but not least their high toughness [7,8], which all contribute to a higher laser damage threshold. This has been shown for Yb^3+^ doped aluminosilicate glasses as compared to phosphate or fluoride phosphate glasses and even to monocrystalline CaF_2_ [9]. Recently, a number of systematic studies on the compositional effects on the optical properties of Sm^3+^ [10,11,12], Eu^3+^ [12,13], Dy^3+^ [14], Tb^3+^ [15], Er^3+^ [16], and Yb^3+^ [9,17] doped aluminosilicate glasses were reported. It has been shown that in addition to the OH concentration, the base glass composition also has a great influence on the optical properties, such as luminescence lifetime and spectral shape of absorption and emission. Particularly noteworthy is the finding that the luminescence lifetimes and the intensity of the so-called hypersensitive transitions in peralkaline and metaluminous aluminosilicate glasses depend differently on the chemical composition. The luminescence lifetime decreases with increasing network modifier oxide (NMO) concentration, i.e., with increasing molecular weight, density, and refractive index of the glass composition, in metaluminous glasses, i.e., where the molar concentration of NMO and Al_2_O_3_ are equal, but increases with increasing NMO concentration in many, although not all, peralkaline glasses, i.e., where the molar concentration of NMO is higher than that of Al_2_O_3_. This effect has so far been observed for Sm^3+^, Eu^3+^, Dy^3+^ and Tb^3+^ doped glasses [10,11,12,13,14,15].

To understand the influence of composition on the optical properties of doped rare earths, it is necessary to consider the structure of aluminosilicate glasses on the atomic scale, i.e., the neighboring atoms and their coordination. While the general atomic structure of glasses can be investigated using Raman and NMR spectroscopy, the local environment of rare earth ions is difficult to access by experimental methods. In a first approximation, the glasses can be differentiated according to their molar network modifier oxide to aluminum oxide ratio (NMO/Al_2_O_3_). At an NMO/Al_2_O_3_ ratio > 1, aluminum is predominantly incorporated as [AlO_4_]^−^ tetrahedra, if only mono- or bi-valent network modifying ions, i.e., alkaline and alkaline earth ions, are considered [18]. That means aluminum oxide predominantly acts as network former in these glasses, and interconnected corner-sharing [SiO_4_] and [AlO_4_]^−^ tetrahedra form the glass network. The tetrahedra are connected by bridging oxygen ≡Si−O−(Si, Al)≡. According to a basic structural model, the negative charges of the [AlO_4_]^−^ tetrahedra are compensated by the network modifying ions (NM). Excess network modifying ions, e.g., K^+^ or Li^+^ which are not required to compensate the charges of the [AlO_4_]^−^ tetrahedra result in the formation of non-bridging oxygen sites ≡Si−O^−^. At an NMO/Al_2_O_3_ ratio < 1, a part of the alumina is also incorporated as [AlO_4_]^−^ tetrahedra with charge compensated by NM ions. The alumina at concentrations exceeding the molar NMO concentration cannot be incorporated as [AlO_4_]^−^ and hence is incorporated in five- or six-fold coordination [18,19,20,21] or connected to oxygen triclusters [18,21,22]. An oxygen tricluster is a configuration in which one oxygen atom is connected to three network former tetrahedra, leading to a formal positive charge at the oxygen atom. These tetrahedra can be two [SiO_4_] and one [AlO_4_]^−^ or one [SiO_4_] and two [AlO_4_]^−^. In the latter case, an additional network modifier cation is required for charge compensation [18,22]. Oxygen triclusters have so far only been detected in calcium aluminosilicate glasses [23], but they are often reported by computational methods [18,24,25,26,27,28,29]. In glasses with an NMO/Al_2_O_3_ ratio < 1, non-bridging oxygen should not occur, however, NBO are still reported in combination with comparably high concentrations of five- or six-fold aluminum [18,19,21]. According to [30], in peralkaline aluminosilicate glasses (NMO/Al_2_O_3_ > 1) the three-dimensional network of interconnected [SiO_4_] and [AlO_4_]^−^ tetrahedra is interlaced by zones of high network modifier concentration, similar to the modified random network model proposed for binary alkali silicate glasses in [31,32]. This structural model implies a micro-segregation in-network modifier-rich regions, the so called depolymerized zones or percolation channels and regions of high network former concentration [18,30]. For binary alkali silicate glasses, this structural model is supported by diffusion and conductivity measurements [33], however, for aluminosilicate glasses, it is so far only supported by numerous computational investigations [18,26,28]. The size and permeation of these zones are most probably decreased with decreasing NMO/Al_2_O_3_ ratio. As shown, the structure of alkali and alkaline earth aluminosilicate glasses is well understood and reported in detail in the literature. By contrast, the incorporation of rare-earth ions has scarcely been investigated. 

In this work, molecular dynamics (MD) simulations to study the structure of lithium and potassium aluminosilicate glasses doped with Gd_2_O_3_ are reported. These glass systems were chosen because of the large ionic radii and electric field strength difference between the network modifier Li^+^ and K^+^ ions. This also results in a large difference in the luminescence properties of the doped rare-earth ions, as explained earlier. Additionally, the results are compared to simulation results of magnesium and barium aluminosilicate glasses of equimolar compositions reported by our group [26]. This work aims to gain further insight into the structure of the aluminosilicate glass network and to analyze in detail the compositional effect of the network-modifying ions on the local environment of the Gd^3+^ ions in these glasses. 

## 2. Computational Details

All MD simulations employed the LAMMPS (Large-scale Atomic/Molecular Massively Parallel Simulator) program package [34] along with a time step of 1.0 fs. Modeling of the potential energy surface (PES) used the interatomic potential of Pedone et al. [35] with a cutoff for short-range interactions of 15 Å and the particle-particle-particle mesh method (PPPM) for long-rage electrostatic interactions [36]. The functional form of the interatomic potential reads:

(1) $$U(r)=\frac{q_{i}q_{j}}{r}+D_{ij}\left[{\left\{1-e^{-a_{ij}(r-r_{0})}\right\}}^{2}-1\right]+\frac{C_{ij}}{r^{12}}$$

where *q_i_* and *q_j_* are the partial charges of atom *i*, *j* along with the interatomic distance *r*, and the adjustable parameters *D_ij_*, *C_ij_*, *a_ij_*, and *r_0_*. All parameters used are listed in Table 1.

Achieving thorough sampling of the PES by MD simulations is essential for reliable structure predictions. In the case of glasses with low dopant concentration, large unit cells (>10^4^ atoms) are required when using standard simulated annealing approaches [37]. In our previous work [26], an alternative approach has been applied for the structural characterization of rare-earth-doped glasses, the so-called inherent structure (IS) sampling. This simulation procedure allows the use of smaller unit cells for the statistically robust sampling of the atomic medium-range order around rare-earth ions. The IS sampling employs MD simulations above the glass transition temperature to overcome higher energetic barriers of the PES and to facilitate diffusion at the ns timescale. The IS are then obtained from structure optimizations of the MD trajectory. The corresponding IS energy determines the probability that the obtained atomic configuration characterizes a particular basin of the PES [38,39,40]. This allows the approximate calculation of the average, macroscopic glass structure. We demonstrated in our previous work [26] that the IS sampling provides atomic structures in good agreement with both standard simulated annealing approaches and experiments. In this work, the starting point of IS sampling was randomly generated glass models with cubic simulation cells corresponding to experimentally observed mass densities. All initial structures were equilibrated for 6.5 ns at T = 3000K after geometry optimization at a constant volume. The equilibration used the canonical (NVT) ensemble along with the Nosé-Hoover thermostat [41,42]. Next, structures taken every 2 ps from the MD trajectory within the last 6 ns were optimized using constant (zero) pressure conditions. The resulting set of potential energies per atom $e_{\mathrm{IS}}$, of the optimized structures yields the distribution $P\left(e_{\mathrm{IS}},T\right)$, which is equivalent to the probability distribution for an atomic configuration with energy $e_{\mathrm{IS}}$, in the liquid state at temperature T (here 3000K) [38,39,40]. $P\left(e_{\mathrm{IS}},T\right)$ allows the approximate calculation of the ensemble average 〈X〉 over n inherent structures (IS) representing the amorphous state using the weighted average:

(2) $$\langle X\rangle =\overset{n}{\underset{i=1}{\sum }}P_{i}X_{i}$$

along with the probability $P_{i}$ of IS i and its structural features $X_{i}$ such as the radial distribution function. Further details on the IS sampling approach for structure predictions of rare-earth-doped glasses can be found in our previous publication [26].

Four different glass compositions were considered: two glasses in the system Li_2_O/Al_2_O_3_/SiO_2_ and two in the system K_2_O/Al_2_O_3_/SiO_2_. In both systems, glasses with equimolar concentrations of NM_2_O (NM, network modifier = Li, K) and Al_2_O_3_ were investigated (20Li_2_O, 20 Al_2_O_3_, 60 SiO_2,_ and 20K_2_O, 20 Al_2_O_3_, 60 SiO_2_), denoted as 20Li and 20K, respectively. In addition, also glasses with a molar NM_2_O/Al_2_O_3_ ratio of 3 were studied in both systems (30Li_2_O, 10 Al_2_O_3_, 60 SiO_2,_ and 30K_2_O, 10 Al_2_O_3_, 60 SiO_2_), denoted as 30Li and 30K, respectively. Since in experimental investigations very similar correlations of luminescence properties and the glass composition was observed irrespective of the rare earth ion used (Sm^3+^, Eu^3+^, Dy^3+^, Tb^3+^) [10,11,12,13,14,15], Gd^3+^ was chosen as model rare-earth ion because of its similar ionic radius (Sm^3+^ (0.96 Å), Eu^3+^ (0.95 Å), Gd^3+^ (0.94 Å), Tb^3+^ (0.92 Å), Dy^3+^ (0.91 Å) [43]) and the available interatomic potential parameters. All structure models contain approximately 1 mol% Gd_2_O_3_. The chemical compositions are summarized in Table 2.

## 3. Results and Discussion

### 3.1. General Structure

**Figure 1:**
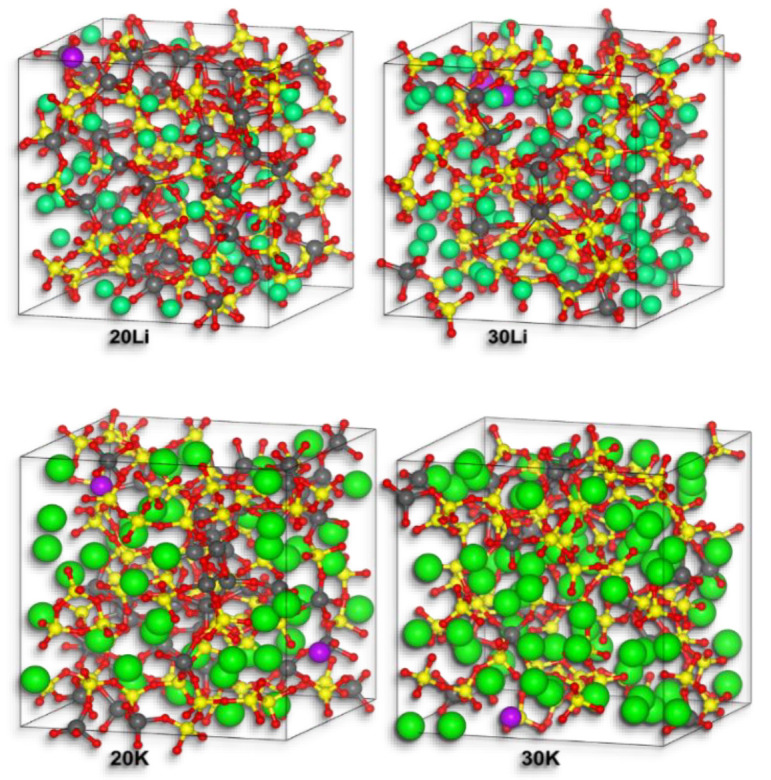
Equilibrium atomic configurations obtained for the four different alkali aluminosilicate glass systems. Si atoms: yellow, Al: grey, O: red, network modifier atoms (Li, K): green, Gd: purple.

Figure 1 shows the equilibrium atomic configurations obtained for the four different alkali aluminosilicate glass systems. In all cases, the systems are composed of an aluminosilicate network with tetrahedrally coordinated Si and Al atoms connected by bridging oxygen atoms. Both types of alkali cations, Li^+^ and K^+^, are located in the gaps or voids of the network structure. The Gd^3+^ ions are located at similar positions as the alkali ions.

According to the modified Random Network model [31,32], percolation channels will be formed if the concentration of NM ions exceeds the percolation threshold. As illustrated in Figure 1, an increase in the NM_2_O/Al_2_O_3_ ratio (20Li and 20K: 1:1; 30Li and 30K: 3:1) increases the number of continuous channels formed by the NM ions. 

**Figure 2:**
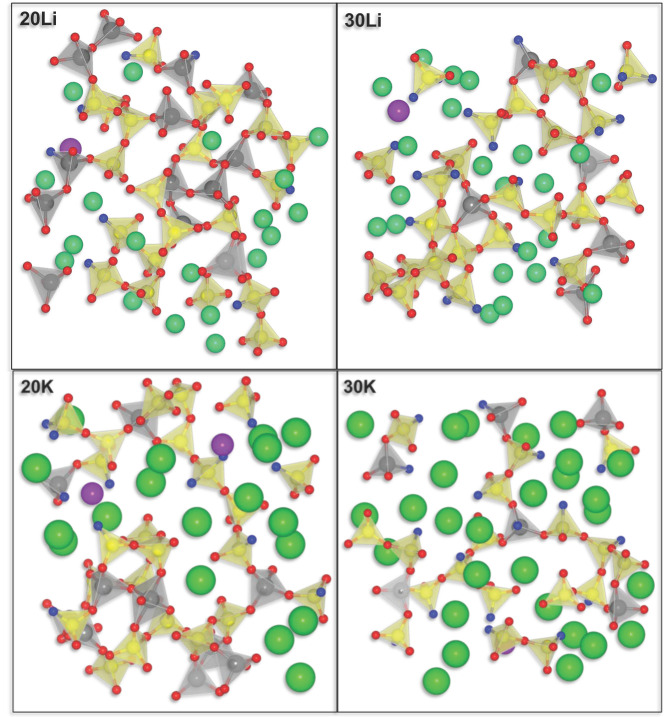
Schematic representation of alkali aluminosilicate glass network fragments comprising [SiO_4_] (yellow) and [AlO_4_]^−^ tetrahedra (grey) as network formers, bridging (red) and non-bridging (blue) oxygen atoms and network modifier ions Li^+^ and K^+^ (green) and Gd^3+^ (purple).

For clarity, Figure 2 shows slice cuts from the structures depicted in Figure 1. The chains of interconnected [AlO_4_]^−^ and [SiO_4_] tetrahedra are clearly visible. In this figure also bridging oxygen (red) and non-bridging oxygen atoms (blue) are distinguished. From these figures, it can already be deduced that, according to the MD simulations, non-bridging oxygen sites exist even in the metaluminous glasses 20Li and 20K, and that the alkali ions occupy regions with a locally increased concentration of network modifying ions, the so-called depolymerized regions or percolation channels. This has already been shown by other studies [44,45]. Note that there are additional structural elements behind and in front of these panes that are not visible but contribute to charge compensation.

**Figure 3:**
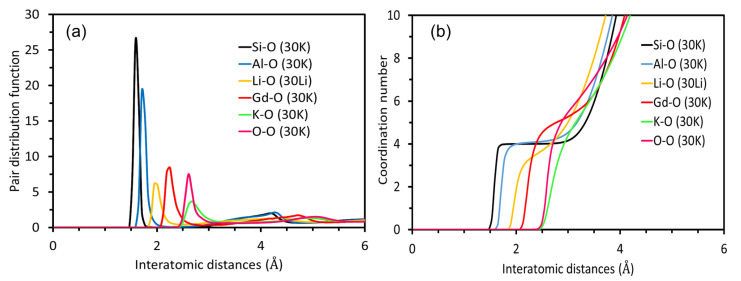
The radial distribution functions (**a**) and the coordination numbers (**b**) of the different atom pairs in the peralkaline alkali aluminosilicate glasses 30K and 30Li.

Figure 3 shows selected radial distribution functions (RDF) as well as coordination numbers (CN) as a function of the distance from the cation in the peralkaline aluminosilicate system 30K. The first maximum of the RDFs in Figure 3a represents the average cation-oxygen bond length. For comparison, the RDF of the Li–O pair in the 30Li glass is added. The RDFs of the other cations in the 30Li composition is very similar to their RDFs in 30K and therefore not shown. In the investigated aluminosilicate glass systems 30K and 30Li, the bond length distribution cation-oxygen is increasing in the order Si–O < Al–O < Li–O < Gd–O < K–O. This result basically complies to the ionic radii of the cations: Si^4+^ (0.40 Å), Al^3+^ (0.54 Å), Li^+^ (0.76 Å), Gd^3+^ (0.94 Å), K^+^ (1.38Å) [29]. For the metaluminous compositions, 20Li and 20K very similar results are found (not shown). The ordinate of the diagram in Figure 3b represents the average coordination number of the respective cations. The coordination number of the Si–O bond is basically constant at four up to a distance of about 3 Å, while the Al–O CN curve is not as flat as that of Si–O, which shows that Al also occurs in higher coordination numbers, such as 5 and 6, as will be discussed later on. 

### 3.2. Structural Influence of the Network Modifier Ions

Table 3 shows the interatomic distances and the coordination numbers (CN) of the network modifier (NM) ions in their first coordination sphere (with oxygen) as well as of their second coordination sphere (with cations). In the case of 20Li and 20K, the average calculated CNs are 3.7 and 6.6, while the average calculated Li–O and K–O bond lengths are 2.01 and 2.67 Å, respectively. These values correspond to the peak positions of the RDFs in Figure 3. The larger ionic radius of K^+^ results in longer bond lengths and higher CNs in the first and second coordination shell compared to Li^+^. According to the basic structural model for aluminosilicate glasses explained in the introduction, the [AlO_4_]^−^ tetrahedra require a cation for charge compensation. Therefore, the distances Li-Al and K-Al are also meaningful, they are 3.03 and 3.54 Å and the calculated CNs are 2.5 and 2.7, respectively. These numbers show that there is no exact one-to-one coordination of NM ions and [AlO_4_]^−^ units. On average, however, the total systems are charge neutral. 

For the peralkaline models 30Li and 30K, the Li-O and K-O bond lengths are 1.95 Å and 2.67 Å, respectively, almost identical to those obtained for the metaluminous compositions 20Li and 20K (2.01 and 2.67 Å). The calculated CNs of the peralkaline compositions 30Li and 30K (3.7 and 6.6) are also similar to those of the 20Li and 20K (3.7 and 6.4). The slightly lower CN for 30K in comparison to that of 20K is within the uncertainty of the simulation method.

Table 3 shows the CNs of the NM ions for the second coordination sphere in all compositions. The CNs with other cations of the peralkaline glasses (30Li, 30K), differ substantially from those of the metaluminous compositions (20Li, 20K). As expected from the chemical compositions with an NM_2_O/Al_2_O_3_ ratio of 3:1, in 30Li and 30K, all [AlO_4_]^−^ tetrahedra should be coordinated by NM ions, however, only a maximum of ⅓ of all NM ions is needed for the charge compensation of the [AlO_4_]^−^ tetrahedra in these glasses.

Hence, the coordination of NM ions with [AlO_x_]^−^ polyhedra is much less likely in the peralkaline glasses than in metaluminous compositions (cf. Table 3). However, the most numerous partners in the second coordination shell of the NM ions are [SiO_4_] tetrahedra.

**Figure 4:**
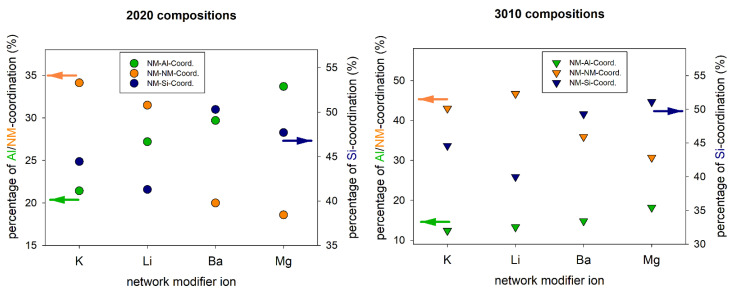
Network modifier-cation coordination fractions for metaluminous (**left**) and peralkaline (**right**) alkali and alkaline earth aluminosilicate glasses. Values for Ba and Mg-containing glasses are taken from [26].

From the absolute coordination numbers, it is difficult to identify any systematical changes in the structure of the glasses with varying composition and network modifier ions. Therefore, the fractions of the NM-cation coordinations in the second coordination sphere were calculated for all compositions (cf. Table 3). Figure 4 shows the fraction of NM-cation coordinations in the metaluminous and peralkaline compositions in comparison to the results obtained in alkaline earth, i.e., in magnesium and barium aluminosilicate glasses of equimolar compositions [26]. In both compositional series (metaluminous and peralkaline) the coordination with other network modifying ions is notably higher for the alkali ions. This finding is trivial since the alkali aluminosilicate glasses have twice as many NM ions due to their chemical composition (e.g., Li_2_O vs. MgO). The opposite trend is observed for the NM-Si coordinations in both series, i.e., the alkaline earth ions have a higher percentage of Si atoms in their second coordination sphere than the alkali ions. For the NM-Al coordinations, it is found that the alkaline earth ions generally have a higher percentage of Al atoms in their second coordination sphere, most likely because two [AlO_4_]^−^ groups are needed for charge compensation. Additionally, the percentage of NM-Al coordinations is higher for smaller NM ions (Li^+^, Mg^2+^). This is probably a result of the generally lower overall coordination numbers for smaller ions. Therefore, at about constant absolute NM-Al coordination numbers (20Li/20K: 2.5/2.7, 30Li/30K: 1.4/1.5) their percentage is higher for smaller ions. For the NM-Si and NM-NM coordinations, no clear correlation with the size of the NM ions can be deduced. However, it must be noted that the NM-NM fractions are higher than the average NM fractions of all cations according to their molar composition (28.6% in metaluminous, 42.9% in peralkaline) (cf. Table 3). The same effect is also observed for alkaline earth aluminosilicate glasses investigated in [26]. This shows that there is a general preference for NM-NM coordinations, even in metaluminous glasses, which corresponds to the formation of depolymerized regions in the glass structure, as seen in Figure 2.

In the following paragraph, the coordination with oxygen atoms in the first coordination sphere is investigated in more detail. The network structure of the considered aluminosilicate glasses consists of [SiO_4_] and [AlO_4_]^−^ tetrahedra connected by bridging oxygen (BO), as shown in Figure 2. Generally, the bridging oxygen can be classified into three different types depending on the tetrahedral type: Si-O-Si, Si-O-Al, and Al-O-Al. In the latter case, the Löwenstein rule is violated, which, however, has already been reported in the literature, e.g., for Li_2_O/Al_2_O_3_/SiO_2_ [46], Na_2_O/Al_2_O_3_/SiO_2_ [47], and CaO/Al_2_O_3_/SiO_2_ glasses [47]. Non-bridging oxygen (NBO), conversely, refers to oxygen atoms that are bonded to only one [SiO_4_] or [AlO_4_]^−^ tetrahedron. As a result, in principle, there are only two types of non-bridging oxygen atoms: ≡Si–O^−^ and ≡Al–O^−^. The latter should be very unlikely because of the repelling force of the two negative charges at different positions in the [AlO_3_O^−^]^−^ group. However, NBO at [AlO_4_]^−^ groups are reported in the literature for calcium aluminosilicate glasses with low SiO_2_ concentrations [48]. As seen in Figure 2, according to the simulation results sporadic NBO at [AlO_4_]^−^ groups can be found. In addition, oxygen may occur in triclusters (Tri), a specific type of oxygen that connects three tetrahedra, as explained in the introduction section.

**Figure 5:**
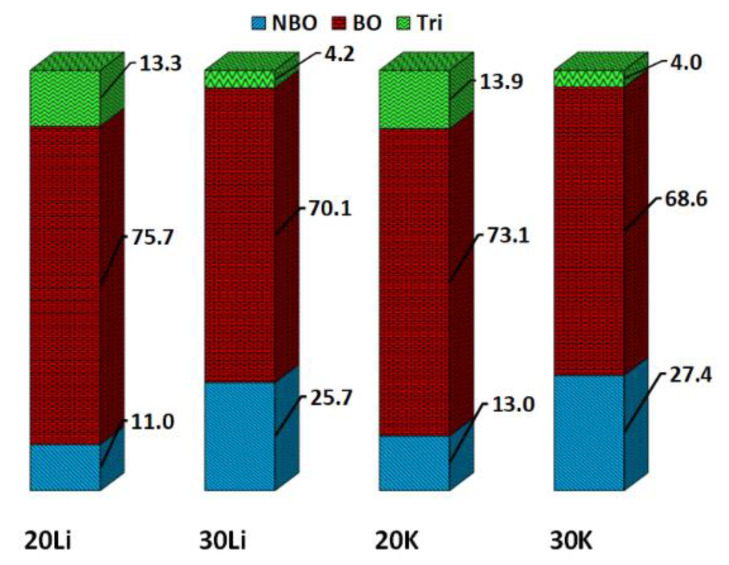
Overall fractions of non-bridging oxygen (NBO), bridging oxygen (BO), and tri-cluster oxygen ions (Tri) in the different chemical compositions.

The fractions of different types of oxygen atoms are shown in Figure 5. As the ratio of NM_2_O/Al_2_O_3_ increases, the number of BO decreases, and the fraction of NBO increases. Generally, the degree of polymerization (DOP) can be defined as the ratio of bridging oxygen to non-bridging oxygen. Therefore, it can be concluded that the DOP shows a declining trend with an increasing NM_2_O/Al_2_O_3_ ratio. The increasing number of network modifier ions (Li^+^/K^+^) in the glass compositions results in depolymerization of the network structures by transforming the BO to NBO, thereby reducing the DOP. In addition, the absolute number of NBO is generally slightly higher in the potassium-containing glasses, probably due to the higher ionic radius of K^+^ and its lower electronegativity. K^+^ should therefore have a higher tendency to disrupt the glass network. The differences in the overall NBO fractions between compositions of constant NM concentration depicted in Figure 5 are not reflected in Table 4. While the coordination with NBO is generally higher for the peralkaline compositions 30Li and 30K due to the higher NM_2_O/Al_2_O_3_ ratio, the data also shows that the coordination numbers with NBO are about the same for Li^+^ and K^+^ ions despite the much higher ionic radius of K^+^, similarly to the coordination with Al given in Table 3. Moreover, the CN with NBO is on average higher than 1 for both ions and both NM_2_O/Al_2_O_3_ ratios, again showing that there is no exact one-to-one charge compensation. However, this is a result of NBO (and [AlO_4_]^−^) counted multiple times for each NM ion. 

**Figure 6:**
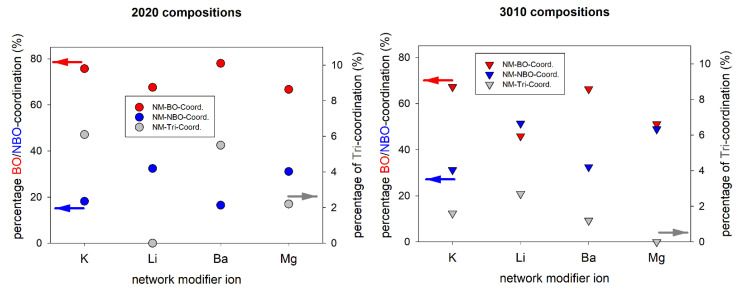
Network modifier-oxygen coordination fractions for metaluminous (**left**) and peralkaline (**right**) alkali and alkaline earth aluminosilicate glasses. Values for Ba and Mg-containing glasses are taken from [26].

This implies that there must be other NM ions close by contributing to the NBO charge compensation, which is another hint at the formation of depolymerized regions in all investigated glass compositions. In peralkaline samples 30Li and 30K, the CNs with NBO and the fractions of NBO are higher than in the metaluminous glasses 20Li and 20K, which shows that the depolymerized regions must be larger in the peralkaline compositions. Compared to the alkaline earth aluminosilicate glasses of equimolar compositions reported before, the coordination numbers with NBO are very similar, i.e., only slightly lower (20Mg: 1.4, 20Ba: 1.5, 30Mg: 2.3, 30Ba: 2.7 [26]). The much higher CN for barium aluminosilicate glasses is probably caused by the higher ionic radius compared to the other NM ions (Li^+^: 0.76 Å, K^+^: 1.38 Å, Mg^2+^: 0.72 Å, Ba^2+^: 1.42 Å), or due to the uncertainty of the simulation method. However, the percentage of NBO coordinations is almost independent of the charge of the NM ion (cf. Figure 6). The percentages of the BO coordinations show the opposite trend compared to the NBO coordinations (cf. Figure 6).

In the case of the calcium aluminosilicate glasses 20Ca and 30Ca (in analogy to the glass designations used here), High-Resolution Magic Angle Spinning Nuclear Magnetic Resonance (HRMAS NMR) investigations found concentrations of up to 5% NBO for metaluminous calcium aluminosilicate glasses [20,21,49] and 23% NBO for peralkaline compositions [50]. In particular, the latter is close to our simulation results of about 25.7% (30Li) to 27.4% (30K). Atila et al. report NBO concentrations of about 7% and 9% in metaluminous lithium and potassium aluminosilicate glasses, respectively, by MD simulations [25]. Similar to our results, they also found a slightly higher NBO concentration for the potassium-containing glass. For metaluminous alkaline earth compositions, they report a clear trend to lower NBO concentrations with increasing NM radii [25]. This effect is also found in our previous publication: (20Mg: 12.4%, 20Ba: 11.1% [26]). Carpentier et al. found NBO concentrations of about 10% and 25% for metaluminous and peralkaline strontium aluminosilicate glasses, respectively, of similar compositions by MD simulations [27]. Lodesani et al. report NBO concentrations of between 16% and 19% for peralkaline sodium and potassium aluminosilicate glasses [24], however, with a lower NMO/Al_2_O_3_ ratio. Generally, they also report higher NBO concentrations for the potassium aluminosilicate glass. Xiang et al. report NBO concentrations of around 10% for a peralkaline sodium aluminosilicate glass and about 4% for a metaluminous sodium aluminosilicate glass [42]. Sadat et al. report NBO concentrations between 2.0 and 3.6% in metaluminous sodium aluminosilicate glasses [51]. It has also been reported that the overall NBO concentration is virtually independent of the used NM oxide [19]. This is different from the results presented here. 

Figure 5 and Figure 6 additionally provide information about the number of oxygen triclusters. With decreasing concentration of NM ions, i.e., decreasing NM_2_O/Al_2_O_3_ ratio, a considerable number of NBO turns into oxygen triclusters while the number of BO increases only slightly. The formation of oxygen triclusters is reported in many publications that use MD simulations for glass structure investigations. Reported are e.g., concentrations of about 10% in metaluminous alkali and alkaline earth aluminosilicate glasses [25], between 0 and 4% for peralkaline alkali aluminosilicate glasses [24], about 5–10% in metaluminous and around 0% in peralkaline strontium aluminosilicate glasses [27], and about 1–1.5% in peralkaline calcium aluminosilicate glasses [29]. However, the calculated tricluster concentration is strongly dependent on the applied interatomic potential [24,27]. Compared to these results, it must be noted that the number of oxygen triclusters is slightly overestimated by the employed structure prediction procedure.

Since an appreciable amount (11.0 to 13.0%) of NBO is present in the metaluminous glass compositions 20Li and 20K, the charge compensation in these glasses must be achieved by aluminum in coordination numbers higher than 4. Table 5 shows the fractions of [AlO_x_] polyhedra present in the four different glass compositions. Generally, the peralkaline glasses show lower concentrations of five- and six-fold coordinated aluminum than the metaluminous compositions. In the glasses with alkali ions of lower ionic radii (20Li and 30Li), the number of higher coordinated Al is notably increased compared to glasses with network modifying ions of larger radius (20K and 30K). This effect has also been observed by ^27^Al NMR investigations [18,19,52], and by MD simulations [25,26].

According to the basic glass structure model, the metaluminous glasses should be perfectly polymerized and all charges of the network modifying ions should be compensated by [AlO_4_]^−^ tetrahedra. Therefore, higher concentrations of five- and six-fold coordinated Al should not at all occur in metaluminous and peralkaline aluminosilicate glasses. However, it is well known that minor concentrations of five- and six-fold coordinated Al actually were found in metaluminous and even, to a lesser extent, in peralkaline aluminosilicate glass [18,19,20,28,49,50]. Also, MD simulations of metaluminous alkali aluminosilicate glasses show about 2–4% [AlO_5_]^2−^ [25]. Sadat et al. found [AlO_5_]^2−^ fractions between 2.4 and 14% in metaluminous sodium aluminosilicate glasses with increasing fractions for higher Al_2_O_3_ concentrations [51]. For peralkaline alkali aluminosilicate compositions about 6% or <1% [AlO_5_]^2−^ depending on the interatomic potential used are reported in [42] and values close to 0% in [25,53]. Considering these values, the number of higher coordinated Al is presumably overrepresented by our simulation results.

Furthermore, the Q_n_ distributions characterizing the [SiO_4_] glass network connectivity were calculated (Table 6). The Q_n_ distributions show a clear difference between metaluminous and peralkaline glasses. In metaluminous glasses, most of the [SiO_4_] groups are Q_3_ and Q_4_ with percentages of 26.0% and 70.9%, respectively, for 20Li and 28.0% and 66.6%, respectively, for 20K. Only very few [SiO_4_] groups exist as Q_2_ and Q_1_. Q_0_ groups are practically non-existent. As already deduced from the overall fractions of NBO in Figure 5, the potassium-containing glass 20K has, according to the simulation results, a more depolymerized network than the 20Li glass. The peralkaline glasses show a broader distribution of Q_n_ groups, 12.4%, 41.5%, and 44.6% occur as Q_2_, Q_3_, and Q_4_, respectively for 30Li and 14.4%, 40.6%, and 42.5% occur as Q_2_, Q_3_, and Q_4_, respectively for 30K. In analogy for the peralkaline compositions, the potassium-containing glass is more depolymerized than its lithium-containing counterpart. This is consistent with the overall fractions of NBO (see Figure 6).

To summarize, the general structure obtained by the IS sampling method is in good agreement with experimental investigations and recent MD simulation results. Especially the influence of the network modifier field strength is well reflected. However, the number of structural elements of high potential energy, such as 5- and 6-fold coordinated aluminum, oxygen triclusters, and probably also the concentration of NBO are overrepresented by this method. 

Further characterization of the glass network and topology can be obtained by, e.g., graph theory [54] to quantify the network connectivity and degree of disorder and will likely be the subject of future studies.

### 3.3. Local Environment around Gd^3+^ Ions

In Table 7, the coordination numbers (CN) of the Gd^3+^ ions in the first coordination sphere (oxygen) and in the second coordination sphere (cations) are summarized for all glass compositions. The Gd-O CNs do not vary much and are between 5.2 and 5.8. These are about the same values that were found for the alkaline earth aluminosilicate glasses (CNs 5.4 to 5.8) [26]. The Gd-O CNs are somewhat smaller in the glasses with NM ions of larger ionic radii (K^+^, Ba^2+^ [26]) than in the glasses with smaller NM ions (Li^+^ and Mg^2+^ [26]). The distance to directly neighboring oxygen atoms is constant at about 2.2 Å for all four compositions. For the second coordination sphere, a different dependency is found: the Gd coordination spheres are notably larger (higher overall CN) in peralkaline glasses (higher NM concentration) and glasses with NM ions of higher ionic radii. This finding shows that the Gd^3+^ ions must be closely coordinated with the NM ions. The Gd-NM CNs show exactly the same trend; they increase in the order 20Li < 20K < 30Li < 30K. This is in analogy to the simulation results for alkaline earth aluminosilicate glasses (20Mg < 20Ba < 30Mg < 30Ba) [26]. The Gd-Al CNs are lower in the peralkaline compositions 30Li and 30K, due to the lower overall Al concentration in these glasses. The Gd-Si CNs show the opposite trend. Obviously, [SiO_4_] groups partially substitute the [AlO_x_] groups in the second coordination shell of Gd^3+^ in the peralkaline compositions. 

**Figure 7:**
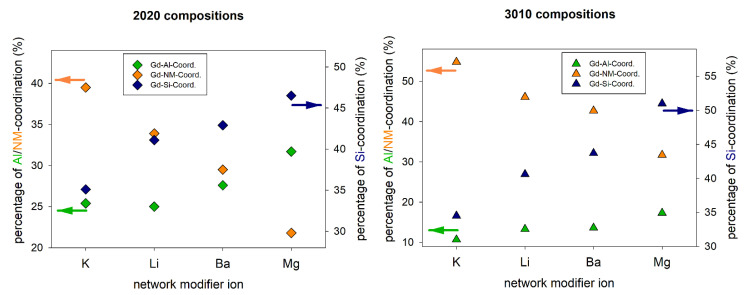
Gd-cation coordination fractions for metaluminous (**left**) and peralkaline (**right**) alkali and alkaline earth aluminosilicate glasses. Values for Ba and Mg-containing glasses are taken from [26].

Figure 7 shows, similarly to Figure 4, the Gd-cation coordination fractions as a function of the network modifier ion for metaluminous and peralkaline alkali and alkaline earth aluminosilicate glasses. Again, the Gd-NM coordination fraction is generally higher in the alkali aluminosilicate glasses, because of their higher NM concentrations. For both glass series (metaluminous and peralkaline) the coordination with NM ions decreases with increasing field strength of the NM ion in both glass types (alkali and alkaline earth). That means the Gd^3+^ ions compete with the NM ions for advantageous positions in the glass network. This competition is, as expected, stronger for NM ions of higher field strengths. The coordination fractions for Al and Si show the opposite effect, however, also here it must be considered that the total number of cations is higher in the alkali aluminosilicate glasses. Therefore, the Gd-Si and Gd-Al coordination fractions must be higher in alkaline earth aluminosilicate glasses compared to the alkali-containing glasses. 

The Gd-Gd CNs are also shown in Table 7. They are lower than 0.1 for all four glasses showing that, on average, less than 10% of the Gd^3+^ ions of the inherent structures form Gd–O–Gd contacts. This indicates a low degree of clustering of Gd^3+^ ions in the investigated aluminosilicate glasses. Therefore, the formation of larger gadolinium clusters appears to be unlikely. However, the Gd-Gd CNs appear to be somewhat smaller for the potassium-containing glasses, probably because of the higher ionic radius of K^+^.

In analogy to Table 4, Table 8 shows the fractions of different types of oxygen in the first coordination sphere of the Gd^3+^ ions. Generally, the CNs with non-bridging oxygen atoms (NBO) is higher in the peralkaline compositions 30Li and 30K. This reflects the higher NM_2_O/Al_2_O_3_ ratio in these glasses. Compared to the NBO coordinations of the network modifier ions in Table 5, the Gd-NBO CNs are much higher, presumably due to the higher charge of the Gd^3+^ ion (charge compensation). Interestingly, the NBO coordination seems to be independent of the network modifier ion. The absolute Gd-NBO CNs are about 3 in all metaluminous and about 4 in all peralkaline compositions, even in the alkaline earth aluminosilicate glasses that were investigated earlier [26]. The CNs with bridging oxygen show the opposite trends, however, this is trivial since the overall Gd-O CNs do not change much (Table 8). The CNs with oxygen triclusters are close to zero for all glasses.

**Figure 8:**
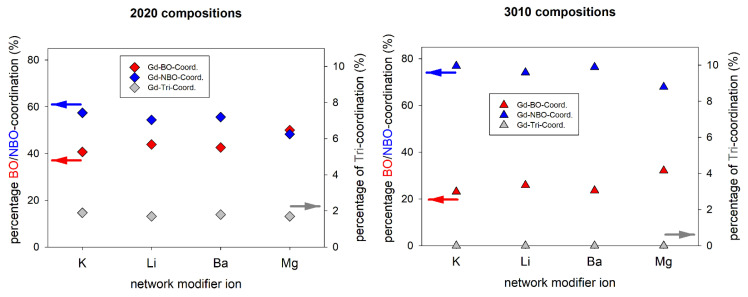
Gd-oxygen coordination fractions for metaluminous (**left**) and peralkaline (**right**) alkali and alkaline earth aluminosilicate glasses. Values for Ba and Mg-containing glasses are taken from [26].

Figure 8 shows the fractions of different Gd-oxygen coordination in metaluminous and peralkaline aluminosilicate glasses depending on their network modifier ions. Although the changes are comparably small, there is a clear trend: the fraction of Gd-NBO coordination decreases with increasing field strength of the NM ion (K^+^ < Ba^2+^ < Li^+^ < Mg^2+^). This trend is observed for both series, metaluminous and peralkaline glasses. The effect of Mg^2+^ ions is particularly strong, as it has by far the highest field strength and is the only one of the four NM ions to have a higher field strength than Gd^3+^. This means that the NM ions (including Gd^3+^) compete for network positions close to non-bridging oxygen sites. In other words, the lower the field strength of the competing NM ions, the more likely are Gd-NBO coordinations. In addition, an increasing NM/Al ratio increases the probability of high Gd-NBO coordination numbers due to the increasing number of NBO in the glass. Accordingly, decreasing CNs with NBO should result in higher CNs with [AlO_4_]^−^ groups because charge compensation must be accomplished. While this effect can be observed for both the alkaline earth series and the peralkaline alkali series (Figure 7), it must be noted that a more general conclusion including alkali and alkaline earth aluminosilicate glasses is difficult due to the different overall numbers of ions in the simulated inherent structures. Therefore, the fractions of Gd-Al coordination of alkali and alkaline earth aluminosilicate glasses in Figure 7 are not comparable.

### 3.4. Correlation of Rare Earth Luminescence and Gd^3+^ Coordination

Detailed investigations on the influence of glass composition on the absorption and luminescence of doped rare-earth ions are very rare. In previous studies, a strong influence of the network modifier ions on the peak splitting of absorption peaks (Er^3+^ in this case) [16] and luminescence emission peaks (Tb^3+^ in this case) [15] and both in the case of Yb^3+^ [17] was reported. A stronger peak splitting was found for glasses with higher NMO/Al_2_O_3_ ratios and glasses with NM ions of lower field strength. These observations correlate very well with the fraction of Gd-NBO coordinations reported here. That means a higher local field strength (and higher splitting of absorption and emission peaks) is most probably related to higher fractions of NBO in the first coordination sphere of the doped rare-earth ions. In [15] also a concentration quenching decreasing effect of NM ions of larger ionic radii is observed. Also, this effect is reflected well by the simulation results. However, the large differences in the luminescence lifetime behavior between peralkaline and metaluminous aluminosilicate glasses described in the introduction section cannot be explained by the simulation results. Probably more advanced simulation methods are needed to get insight into the spatial electron distributions and densities around the rare-earth ions. 

## 4. Conclusions

In summary, the effect of the network modifier ion and the network-modifier oxide to Al_2_O_3_ ratio on the atomic medium-range structure of the Gd^3+^ ions in Gd_2_O_3_ doped alkali aluminosilicate glasses containing K_2_O and Li_2_O was investigated by using molecular dynamics simulations. For this, a computationally efficient simulation procedure, the inherent structure sampling was used. The general structure of the glasses, especially the effect of the different network modifier ions is in very good agreement with recent studies of different groups. However, the number of structural elements of high potential energy, such as 5- and 6-fold coordinated aluminum, oxygen triclusters, and probably also the concentration of non-bridging oxygen (NBO) are overrepresented by this method.

The Gd^3+^ ions are similarly incorporated into the glass structure as the network modifying ions. In the investigated compositions they are closely coordinated with the network modifying ions and form so called depolymerized regions. There is a clear tendency for increasing concentrations of NBO atoms in the coordination sphere of the doped Gd^3+^ ions with decreasing field strength and increasing concentration of the network-modifying ions in the glass composition. It could be shown that this result is not only valid for the alkali aluminosilicate glasses that were investigated here, but also for alkaline earth aluminosilicate glasses of equimolar compositions from an earlier investigation. Probably higher coordination with NBO is the cause of more split absorption and luminescence emission spectra of rare-earth-doped aluminosilicate glasses. In addition, a low tendency for the formation of Gd–O–Gd contacts is found. Here, network modifying ions of larger ionic radii reduce the probability of Gd–O–Gd contacts, which should decrease the luminescence concentration quenching. 

It was also shown that different network-modifying ions are incorporated into the glass structure in different ways depending on their radius and charge, i.e., their field strength. Larger ions have higher coordination numbers and higher distances to their neighboring atoms. However, smaller and higher charged network-modifier ions prefer the coordination with non-bridging oxygen sites. In other words, high field strength network-modifying ions compete with other high field strength ions, such as Gd^3+^ and probably other rare-earth ions, for positions of high NBO coordination and therefore force the Gd^3+^ ions into less advantageous positions with lower non-bridging oxygen coordination. On the other hand, the NBO coordination numbers of Gd^3+^ are notably increased in glasses with network modifier ions of low field strength. This effect could be exploited to tailor the optical properties of the doped ions and to develop new optically active materials.

## Figures and Tables

**Table 1:** Potential Parameters used for the MD simulations. Adapted from [35].

Pair	*D_ij_* (eV)	*a_ij_* (Å^−1^)	*r_0_* (Å)	*C_ij_* (eV Å^12^)
Li^0.6^–O^−1.2^	0.001114	3.429506	2.681360	1.0
K^0.6^–O^−1.2^	0.011612	2.062605	3.305308	5.0
Si^2.4^–O^−1.2^	0.340554	2.006700	2.100000	1.0
Al^1.8^–O^−1.2^	0.361581	1.900442	2.164818	0.9
Gd^1.8^–O^−1.2^	0.000132	2.013000	4.351589	3.0
O^−1.2^–O^−1.2^	0.042395	1.379316	3.618701	22.0

**Table 2:** Unit cell compositions of the glass structure models.

		Chemical Composition [mol%]			
**Model**	**Unit Cell**	**NM_2_O**	**Al_2_O_3_**	**SiO_2_**	**Gd_2_O_3_**
20Li	Gd_2_Li_50_Al_50_Si_75_O_253_	19.8	19.8	59.5	0.9
30Li	Gd_2_Li_75_Al_25_Si_75_O_228_	29.8	9.9	59.5	0.8
20K	Gd_2_K_50_Al_50_Si_75_O_253_	19.8	19.8	59.5	0.9
30K	Gd_2_K_75_Al_25_Si_75_O_228_	29.8	9.9	59.5	0.8

**Table 3:** Coordination numbers (CN) and interatomic distances of the network modifier (NM) ions in the first coordination shell (oxygen) and the second coordination shell obtained for different chemical compositions. For the second coordination shell, the fractions of the different cation coordination are additionally given.

CN Fraction (Distance)	20Li	30Li	20K	30K
NM-O	3.7	3.7	6.6	6.4
	(2.01 Å)	(1.95 Å)	(2.67 Å)	(2.67 Å)
NM-NM	2.9	4.9	4.3	5.2
	31.5%	46.7%	34.1%	43.0%
	(2.61 Å)	(2.61 Å)	(3.39 Å)	(3.21 Å)
NM-Al	2.5	1.4	2.7	1.5
	27.2%	13.3%	21.4%	12.4%
	(3.03 Å)	(3.09 Å)	(3.54 Å)	(3.39 Å)
NM-Si	3.8	4.2	5.6	5.4
	41.3%	40.0%	44.4%	44.6%
	(3.15 Å)	(3.0 9Å)	(3.39 Å)	(3.45 Å)
ΣCN	9.2	10.5	12.6	12.1

**Table 4:** Separation of NM-O coordination numbers (CN) into non-bridging oxygen (NBO), bridging oxygen (BO), and tri-cluster oxygen ions (Tri) and their fractions.

CN Fraction	20Li	30Li	20K	30K
NM-NBO	1.2	1.9	1.2	2.0
	32.4%	51.4%	18.2%	31.2%
NM-BO	2.5	1.7	5	4.3
	67.6%	45.9%	75.7%	67.2%
NM-Tri	0.0	0.1	0.4	0.1
	0.0%	2.7%	6.1%	1.6%

**Table 5:** The fractions of [AlO_x_] groups with three-, four-, five- or six-fold oxygen coordination in the simulated alkali aluminosilicate glasses.

[AlO_x_] Fractions	20Li	30Li	20K	30K
[AlO_3_]	0.7%	0.3%	1.1%	0.8%
[AlO_4_]^−^	78.9%	81.0%	82.8%	91.0%
[AlO_5_]^2−^	18.9%	17.5%	15.0%	7.9%
[AlO_6_]^3−^	1.5%	1.2%	1.1%	0.3%

**Table 6:** The fractions of Si Q_n_ groups in the simulated alkali aluminosilicate glasses.

Q_n_ Fractions	20Li	30Li	20K	30K
n = 0	0.0%	0.1%	0.0%	0.1%
n = 1	0.1%	1.4%	0.4%	2.4%
n = 2	3.0%	12.4%	5.0%	14.4%
n = 3	26.0%	41.5%	28.0%	40.6%
n = 4	70.9%	44.6%	66.6%	42.5%

**Table 7:** Coordination numbers (CN) and interatomic distances of the Gd^3+^ ions in the first coordination shell (oxygen) and the second coordination shell obtained for different chemical compositions. For the second coordination shell, the fractions of the different cation coordinations are additionally given.

CN Fraction (Distance)	20Li	30Li	20K	30K
Gd-O	5.7	5.8	5.4	5.2
	(2.25 Å)	(2.25 Å)	(2.19 Å)	(2.25 Å)
Gd-Al	2.8	1.7	2.9	1.4
	24.8%	13.2%	25.3%	10.7%
	(3.57 Å)	(3.51 Å)	(3.51 Å)	(3.63 Å)
Gd-Si	4.6	5.2	4.0	4.5
	40.7%	40.3%	34.9%	34.3%
	(3.57 Å)	(3.57 Å)	(3.57 Å)	(3.63 Å)
Gd-NM	3.8	5.9	4.5	7.15
	33.7%	45.7%	39.3%	54.5%
	(3.15 Å)	(3.09 Å)	(3.81 Å)	(3.75 Å)
Gd-Gd	0.09	0.10	0.06	0.07
	0.8%	0.8%	0.5%	0.5%
	(4.02 Å)	(3.9 Å)	(3.78 Å)	(4.14 Å)
Σ CN	11.29	12.90	11.46	13.12

**Table 8:** Separation of Gd-O coordination numbers (CN) into non-bridging oxygen (NBO), bridging oxygen (BO), and tri-cluster oxygen ions (Tri) and their fractions.

CN Fraction	20Li	30Li	20K	30K
Gd-NBO	3.1	4.3	3.1	4.0
	54.4%	74.1%	57.4%	76.9%
Gd-BO	2.5	1.5	2.2	1.2
	43.9%	25.9%	40.7%	23.1%
Gd-Tri	0.1	0.0	0.1	0.0
	1.7%	0.0%	1.9%	0.0%

## Data Availability

The data presented in this study are available on request the corresponding author.
